# Future Anxiety Among Young People Affected by War and Armed Conflict: Indicators for Social Work Practice

**DOI:** 10.3389/fsoc.2021.729811

**Published:** 2021-11-29

**Authors:** Nouf M. Alotaibi

**Affiliations:** College of Social Work, Princess Nourah Bint Abdulrahman University, Riyadh, Saudi Arabia

**Keywords:** future anxiety, international students, social work, youth affected by wars and conflicts, social behaivor

## Abstract

Strengthening the evidence base for professional social work intervention that contributes to providing psychosocial support to international students affected by war and conflict is a major priority as this vulnerable group of youth increases. Therefore, this study aimed to determine the level of future anxiety among international students coming from areas experiencing war and conflict. This study used the descriptive correlative approach, where the future anxiety scale was applied to a sample of 287 international students affected by war and conflicts. Findings showed that there are statistically significant differences between males and females (in favor of females) in the level of the social dimension of future anxiety. The current study results showed a statistically significant relationship between future anxiety and some variables related to war and conflict (living in a war environment - direct and indirect exposure to damage). There are statistically significant differences between those who lived in Yemen at the time of wars and those who did not live (in favor of those who lived in Yemen at the time of wars) in the level of future anxiety. There are also statistically significant differences between those exposed to harm or their family because of the war and those who were not exposed (in favor of those who were exposed) in the level of future anxiety as a whole. The study recommends developing psychosocial support services for this vulnerable group, considering the cultural context to promote women and protect them from discrimination in the services they deserve on an equal basis with men.

## Introduction

The world is witnessing a noticeable increase in international migration for higher education, and countries are competing to attract international students and internationalize their educational systems. Undoubtedly, many factors affect students’ choice of the country in which they will receive their education, the most prominent of which is the quality of education in the target country and the possibility of obtaining employment opportunities in addition to the scholarships provided by those countries (Snoubar and Hawal, 2015; Özoğlu et al., 2015; Boyacia and Ozb, 2018). In choosing the country in which they will spend an important part of their life, young international students focus on a set of factors, which are often for future considerations that will help them improve their lives and secure their future. In a study conducted on 367 international students in Turkey to determine their social needs to build a model for the general practice of social work, its results showed that the cultural proximity to the state, scholarships, and the possibility of future work are among the most factors that affected the selection of international students to study in Turkey (Snoubar and Hawal, 2015). Undoubtedly, the impact of these factors extends to the lives of students and their interaction with the social and cultural environment of the host community, which will later shape the cultural experiences of the students themselves, as indicated by the results of the (Newsome and Cooper, 2016), which examined the cultural and social experiences of international students in a British university and those coming From Asian and Middle Eastern countries, international students choose the country based on high expectations. Still, they face culture shock upon arriving in the host country, which may pose future concerns for students.

Considering many variables such as age group, cultural and social background, economic status of international students, language difficulty, housing problems, and establishing social relations, we find many studies (Anjalin et al., 2017; Wang et al., 2017; Hawes, & Thomas, 2018; Gündüz & Alakbarov, 2019; Nilemar & Brown 2019) dealt with the impact of these factors on adaptation and the problems that international students may face in the host country. Its results concluded the importance of these factors and their impact on international students’ adaptation, academic status, and psychological health.

Looking at the previous studies that dealt with international students, we find that they focused on the economic, cultural, academic, psychological, and social conditions of international students in general. We did not find studies examining international students’ problems coming from areas experiencing wars and conflicts, specifically concerning future concerns (Snoubar and Hawal, 2015). As the impact of living in an environment of wars and conflicts puts university students in dire need of sympathy, advice, and assistance, and they need professional interventions in dealing with them (Sengupta, 2017). This is because living in areas witnessing wars and armed conflicts, or the survival of part of the family and relatives in those areas has a significant impact on young people from various psychological and social aspects, in turn, which may affect their adaptation and academic achievement and raise their fear and anxiety over their future. This study focused on Yemeni students to examine problems related to the future anxiety of this group of young people that were not covered by social work studies. Discussing the issues faced by this group can contribute to developing the practice of social work with young victims of wars and conflicts.

The number of Yemeni students in Turkey is constantly increasing, and their number in 2019 reached 3,076 students. Many reasons prompted Yemeni students to choose Turkey for university studies, such as cultural proximity, quality of education, and scholarships for Yemeni students (Altunbas, 2020). However, these international students need to be examined for their psychological and social problems affecting their well-being. The events they witnessed in the war environment, and the difficulties experiences could negatively affect them. A study conducted on Yemeni students in Turkey indicates various forms of prejudice (religious, social, sexual, and political) negatively related to their self-esteem. This poses a real challenge in the adjustment of Yemeni students to the new society and perhaps their outlook on the future (Gawas, 2020).

In general, it can be said that these students have problems that require direct intervention from the social worker, which are in essence different compared to the problems faced by international students coming from countries experiencing stability, security, and peace. Therefore, this study aims to identify future anxiety levels from multiple levels: psychological, economic, health, and social among Yemeni youth coming from war and conflict areas and studying in Turkey.

### The Mental Health of Youth Immigrants Affected by War and Conflict

Psychological problems are prevalent among young people who have lived through wars and conflicts and accompany them post-conflict. Young people in the post-conflict stage need mental health services because of the damage they have sustained due to living in that environment or exposure to traumatic events and experiences, whether directly or indirectly (Newnham et al., 2015). PTSD, anxiety, and depression are among the most common symptoms of war and conflict-affected youth reported by youth (Thabet et al., 2004; Montgomery, 2011; Pfeiffer & Elbert, 2011; Dimitry, 2012; Snoubar & MuSAH, 2017). In general, mental health problems among immigrant youth are considered a challenge because of the challenges they pose to primary care due to cultural and language differences. Also, mental health problems faced by young people are related to the nature of the traumatic experiences they experienced before, during, and after migration. These stressful situations for immigrant youth impact their adaptation and attitudes towards the future (Kirmayer et al., 2011). The traumatic experiences in the environment of conflict and war, in addition to the daily pressures faced by immigrant youth in the host country, lead to an increase in the level of symptoms of anxiety, post-traumatic stress disorder, and depression, which makes this group of young people more vulnerable than others to developing mental health problems (Vervliet et al., 2014). Also, several factors affect the adaptation of immigrant youth, such as turmoil in the family, different culture, the search for identity in the new society, and learning the new culture. Therefore, effective programs are needed to help immigrant youth from diverse cultural backgrounds reduce their fears about the future and facilitate their integration into the new society (Woodgate & Busolo, 2021). Young people who witnessed the events of the conflict need mental health services and appropriate clinical interventions that help them integrate into the new society and eliminate their fears about their future with the continuation of conflicts in their country of origin. On the contrary, the inability to address the mental health needs of these young people is a source of concern indicated by him. Social and mental health service providers working with youth affected by war and conflict (Forrest-Bank et al., 2019). As the resettlement of young people coming from war and conflict areas and helping them to adapt to the countries hosting them is considered a long-term process due to the pre-migration experiences such as the conditions of war and conflicts and the economic and social conditions that they left behind, in addition to negative experiences in the host countries such as racism, discrimination and their lack of acceptance (Rossiter et al., 2015). Which makes them in a state of anxiety and fear for their future in light of the unknown in their country and the new environment that contains many challenges. Therefore, studying the reality of these students and identifying their fears about the future provides useful indicators and helps professional intervention with this group in facilitating their adaptation to the new society and helping them overcome their future fears.

### The Future of Conflict-Affected Youth in the Host Country

The growing crises in many countries of the world, specifically in the regions witnessing wars and conflicts in the Middle East, have affected the future uncertainty for young people due to unemployment and poverty and pushed them to search for a new future through migration to stable countries and European countries (IOM, 2014). Wars and conflicts have clearly affected the increase in problems experienced by young people in the Middle East. In the pre-conflict periods, young people in the Middle East suffered from many economic and social problems that many countries failed to implement social and youth policies to overcome. With the start of armed conflicts and the involvement of young people in them, the problems they face at all levels increased, leading to grave violations of their rights, torture and violence, and their involvement in the conflict. This situation made young Middle Easterners vulnerable to an uncertain future (Snoubar and Hawal, 2015). As the high unemployment among young people and the increase in other problems they suffer from due to wars is one of the most important problems that prevent their ability to foresee the future in countries experiencing wars and conflicts (Messkoub, 2008). Therefore, the future of the immigrant youth group from these regions has high ambitions and hopes for a better future in the host countries. Young people coming from these areas face many challenges in new societies due to the experiences of living in an armed conflict environment, which may negatively affect their integration. In addition, the lack of social support for this group may lead to a deterioration of mental health. Therefore, the host community needs mental health support services as young people’s psychological and social well-being from war and conflict areas are affected by services and psychosocial support programs (Stark et al., 2020). However, these services play a central role in promoting immigrant youth’s mental health and psychosocial well-being, influencing their future orientations (Stark et al., 2021). Because wars and armed conflicts and the long-term damage they cause to people and society are among the most important factors that push young people to think about the future in a way that may differ from immigrant youth who lived in a safe and stable environment, and this may affect their mental health and face many challenges in the new society.

The mysterious events of the future and the fears they cause for students are considered sources of psychological problems they face and form future pressures and anxiety (Al-Otaibi, 2016). Future anxiety makes students feel fear and tension from the events that may happen and in the future and pose a threat to them at the educational, professional, personal, economic, and social levels (Zaleski, 1996). In addition to the impact of future anxiety on pessimism among students, and this is related to the emergence and recurrence of behaviors such as emotions, introversion, hesitation, and doubt, which affects students’ feelings and attitudes and makes them in a negative state and dissatisfaction with oneself (Alshaer & Kaviani, 2019). On the other hand, we find many problems faced by young people coming from war and conflict areas that require interventions at the community level, such as the racial discrimination that awaits them in the communities to which they fled. Young people from the Middle East and North Africa were subjected to harassment and racism, especially after the September 11 attacks (Sutton, 2002). In general, we find that the impact of wars and conflicts on young people has many facets and aspects that accompany them in the war environment and in the countries they seek refuge in, whether as asylum seekers or international students. The continuation of wars in their countries of origin and their problems in the host country may increase their future anxiety and require professional intervention to help this vulnerable group of young people adapt to the new society and plan positively for the future.

### Social Work Practices in Improving the Lives of Young Immigrants

Migration is a state of risk for mental disorders and mental health problems. Its severity increases according to the circumstances in which the migration took place and the problems faced by the immigrant before migration (Potocky & Naseh, 2020). Also, in addition to the traumatic experiences, heavy losses, and difficulties related to mental health that young people face in the war and conflict zone, they face constant pressures within the host country, and this requires awareness of risk and protection factors to develop mental health services and build an appropriate intervention model that helps them get out of the state of distress and forming a positive outlook towards life and the future (Ehnholt and Yule, 2006). In this context, European research and studies indicate the need for social workers to focus on developing skills to work with migrants at all levels, micro, mezzo, and macro, to achieve their well-being and stability in new societies (Nash et al., 2006; Tembo et al., 2021). The challenges faced by immigrant youth and those affected by war and conflict are among the main topics in social work practice. These young people face many challenges before migration and challenges in the host country, such as integration and social exclusion. This requires social work intervention to preserve the well-being of young migrants (Ims et al., 2021). In this context, social workers work to reduce the risks of social exclusion and the potential effects on the mental health of immigrants by strengthening social support services directed at them, whether formal or informal (Sandhu et al., 2013). Therefore, there is an urgent need to learn about the most important psychological and social interventions and therapeutic methods that include effective techniques and strategies that can be useful during professional practice with young people affected by the events of the war. Furthermore, identifying the elements of professional practice of effective interventions may contribute to developing clinical intervention with this vulnerable group of young people (Brown et al., 2017). Social work interventions should focus on the vulnerabilities of conflict-affected youth, and this requires collaborative approaches across responsible bodies and service resources available for youth welfare (Betancourt et al., 2013). The practice of social work, the application of individual and group programs, psychosocial support programs, and a strengths-based flexibility intervention program are among the most important social work interventions with young people coming from war and conflict areas. It enhances many youth skills such as copying, adaptation, relaxation, communication, and problem-solving skills. Also, the intervention at the individual level and the realization of individual sessions contribute to helping young people tell their stories. Interventions with these young people impact acquiring individual skills that help them cope and help them increase knowledge in the cultural and social context of the host country, which may reduce their fears about the future (Crooks et al., 2020). With the increase in the number of international students coming from areas witnessing wars and conflicts, there is an urgent need to build evidence and develop indicators that help social workers to make effective interventions with young people affected by these dangerous events. Furthermore, improving mental health and providing psychological and social support to this vulnerable group of young people through many programs developed by the social worker may enhance their positive outlook on the future.

## The Current Study

This study focused on studying future anxiety among international students affected by wars and conflicts. The main aims of the study are 1) Determining the level of future anxiety of international students coming from areas experiencing war and conflict. 2) Verifying if there are significant differences in future anxiety according to demographic characteristics of international students coming from war and conflict areas. 3) Verifying if there are significant differences in future anxiety according experiences related to the environment of war and conflict. 4) Determining indicators of social work intervention that may help reduce future anxiety among students coming from war and conflict areas. To achieve the research goals, the following hypotheses have been set: 1) There is a significant difference in future anxiety mean and the hypothesized mean (comparison mean). 2) There are significant differences in future anxiety according to demographic characteristics of international students coming from war and conflict areas. 3) There are significant differences in future anxiety according to some variables related to the war and conflict environment (living in a war environment - direct and indirect exposure to damage - continuing family presence in a war and conflict environment).

### Terminology of Study

International students: The students who move from their country of origin to another country, intending to obtain higher education in its various stages (bachelor’s, master’s, and doctoral degrees). This study identifies with Yemeni students who came to Turkey for undergraduate studies and enrolled in the 2020–2021 semester at Karabuk University.

Migration for Education: Yemeni international students migrate to Turkey to obtain higher education for many motives and reasons.

Future anxiety: the score obtained by the international student examined on the future anxiety scale used in this study.

### Methodology

#### Sample

The sampling frame is designed to enumerate all Yemeni international students studying in Turkish universities through the academic year 2020–2021. Due to the many determinants represented in the time allocated for research and the financial cost and the inaccessibility of all students, the researcher is satisfied with studying Yemeni students at Karabuk university, as it is the most Turkish university that receives international students coming from Yemen, according to the statistics of the Turkish Ministry of Higher Education (YÖK, 2021), where the number of Yemeni students registered for the academic year 2020–2021 reached 660 (574 male and 86 female).

The questionnaire was distributed electronically to all 660 Yemeni students studying at the Karabuk University between April 2021 and May 2021. The students were included if 1) they had Yemeni citizenship, 2) they agreed voluntarily to participate in the research after reading informed consent 3) they are actually enrolled in the academic year 2020–2021. The data was collected using the double sampling design to minimize the non-response bias. In phase one, data was collected from the population (660 students) by sending the survey to all Yemeni students. At this stage, 142 students responded to the online survey after several reminders. The response rate for this stage was 21.52%.

Consequently, the population was divided into two subpopulations of 142 responses and 518 non-responses. To gather more information from the non-response subpopulation, a more intensive data collection method such as phone calls and private messages was implemented to gather more information from the non-response subpopulation in phase one to gather more information. A simple random sample of size 250 is selected from the non-response subpopulation. Then the survey was sent to them by email, followed by phone calls and private messages, including several incentives. At this stage, the response rate was approximately 58% (145/250), higher than phase one (21.52%). Finally, out of 660 male and female students, only 287 male and female students responded to the questionnaire according to the specific conditions that the students were informed about. The final response rate was 43.48%, with a margin of sampling error of ±4.35%.

#### Study Materials

This study relied on the future anxiety scale as a tool for collecting data prepared for research purposes by taking the opinions of three faculty members in social work. The Future Anxiety Scale was chosen after the researcher conducted an insightful interview with 10 Yemeni students from a distance using the Microsoft Teams program to identify the most problems facing these students. It was found that the most common problems they face are future anxiety.

The Future Anxiety Scale (Al-Khalidi, 2002) was used, which consists of 48 items, 15 positive items, and 33 negative items, distributed over five dimensions: the psychological dimension, the economic dimension, the social dimension, the health dimension, and the family dimension. It is a measure with a high level of reliability or validity, and 39 phrases have been approved and modified to suit the research topic. In addition to a section dedicated to demographic variables: gender, age, marital status, number of years of residence in Turkey, school year, economic status. In addition to a section dedicated to experiences related to the environment of war and conflict.

#### Procedure

Quantitative research methods were used to collect data from Yemeni students studying at Karabuk University. Questions were developed to collect demographic data and use the Al-Khalidi (2002) Future Anxiety Scale, which consists of 39 phrases, to collect data related to psychological anxiety. Besides emails and phone calls, the questionnaire was distributed to all students electronically through many channels and access to the communication groups, such as WhatsApp groups. Informed consent was placed at the beginning of the questionnaire, which indicates the confidentiality of information, how it is stored and used, and the student’s right to withdraw whenever he wants. The data collection process took 20 days, from April 20, 2021, to May 10, 2021, where 287 male and female students were reached out of 660 students.

#### Methodology Limitations

There is a lack of previous research studies on the future anxiety of young people from war and conflict areas. Also, may the mixed method be more suitable for this kind of study, but the conditions resulting from COVID-19, the precautionary measures, and the pressures students face due to the pandemic were considered when designing the methodology. However, we recommend conducting studies with a mixed method to study future anxiety for international students from war and conflict areas.

#### Data Analysis

SPSS (version 26) was utilized for analysing the survey data. To achieve the study’s aims, some advanced statistical techniques besides the descriptive statistics utilized to study the difference and association, such as; Spearman correlation coefficient, One-sample *t*-test, and independent-sample *t*-test.

## Results

The study results were discussed according to the main hypotheses of the research and were presented in the following tables. Table 1 summarizes the first hypothesis test: There is a significant difference in future anxiety mean and the hypothesized mean (comparison mean).

**TABLE 1:** The below table shows the mean, standard deviation, and result of a one-sample *t*-test using different future anxiety dimensions.

Dimensions	Mean	SD	*t*-value	*p*-value
Psychological	2.71	0.65	−7.54	1.00
Economical	2.84	0.66	−4.05	1.00
Social	3.34	0.61	9.50	0.00
Health	2.80	0.78	−4.40	1.00
Family	3.17	0.54	5.27	0.00
Total Future Anxiety	2.93	0.50	−2.34	0.99

According to the table above, the means of social dimension (*t*-value = 9.50, *p*-value = 0.00) and family dimension (*t*-value = 3.17, *p*-value = 0.00) are significantly greater than three, which is the threshold limit for high future anxiety level.

Tables 2, 3 summarize the second hypothesis test: There are significant differences in future anxiety according to demographic characteristics of international students coming from war and conflict areas.

**TABLE 2:** The below table show relationship between different demographical variables (ordinal variables), and various dimensions of future anxiety, using the Spearman correlation coefficient.

Variables		Correlation Coefficient
Age	Psychological Dimension	0.03
Years of residence in Turkey		0.07
Academic year		0.03
Economic situation		−0.12*
Age	Economical Dimension	−0.01
Years of residence in Turkey		0.08
Academic year		0.09
Economic situation		−0.13*
Age	Social Dimension	0.07
Years of residence in Turkey		−0.05
Academic year		−0.15*
Economic situation		0.02
Age	Health Dimension	−0.02
Years of residence in Turkey		0.03
Academic year		−0.10
Economic situation		−0.12*
Age	Family Dimension	−0.02
Years of residence in Turkey		−0.06
Academic year		−0.12*
Economic situation		−0.12*
Age	Total Future Anxiety	0.01
Years of residence in Turkey		0.03
Academic year		−0.07
Economic situation		−0.10

**TABLE 3:** The below table shows the differences between those who have been married and those who never married before in terms of future anxiety and its dimensions.

Dimensions	Have you ever been married?	N	Mean	SD	*t*-value	*p*-value
Psychological	Yes	44	2.66	0.59	−0.631	0.529
	No	243	2.72	0.66		
Economical	Yes	44	2.61	0.63	−2.547	0.011
	No	243	2.88	0.65		
Social	Yes	44	3.35	0.54	0.082	0.935
	No	243	3.34	0.62		
Health	Yes	44	2.89	0.88	0.825	0.410
	No	243	2.78	0.76		
Family	Yes	44	3.25	0.49	1.059	0.291
	No	243	3.15	0.55		
Total Future Anxiety	Yes	44	2.92	0.49	−0.184	0.854
	No	243	2.93	0.50		

According to the table above, there is a significant difference between males and females in terms of the economic dimension in favor of males (*t*-value = 2.365, *p*-value = 0.019). Moreover, there is significant difference between male and female in term of social dimension in favor of females (*t*-value = −2.743, *p*-value = 0.006). On the other hand, the *t*-test showed insignificant differences between males and females in terms of psychological, Health, Family, and total future anxiety dimensions (*p*-value > 0.05).

According to the table above, there is a significant difference between those who have been married and those who never married before (*t*-value = −2.547, *p*-value = 0.011). The average score for those who never married before was significantly greater than those who have been married. On the other hand, the *t*-test showed insignificant differences between those who have been married and those who never married before in terms of psychological, Social, Health, Family, and total future anxiety dimensions (*p*-value > 0.05).

Tables 4–6 summarize the third hypothesis test: There are significant differences in future anxiety according to some variables related to the war and conflict environment (living in a war environment - direct and indirect exposure to damage - continuing family presence in a war and conflict environment).

**TABLE 4:** The below table shows the differences between those who have lived in Yemen during the war and conflicts and those who haven’t in terms of future anxiety and its dimensions.

Dimensions	Have you lived in Yemen during the war and conflicts?	N	Mean	SD	*t*-value	*p*-value
Psychological	Yes	237	2.74	0.65	1.470	0.143
	No	50	2.59	0.64		
Economical	Yes	237	2.89	0.64	2.775	0.006
	No	50	2.61	0.68		
Social	Yes	237	3.34	0.60	0.152	0.879
	No	50	3.33	0.65		
Health	Yes	237	2.85	0.77	2.623	0.009
	No	50	2.54	0.81		
Family	Yes	237	3.18	0.53	1.167	0.244
	No	50	3.09	0.55		
Total Future Anxiety	Yes	237	2.96	0.49	2.227	0.027
	No	50	2.79	0.52		

**TABLE 5:** The below table shows the differences between those who have been (or their family) exposed to any harm because of the war and those who haven’t, in terms of future anxiety and its dimensions.

Dimensions	Have you or your family been exposed to any harm because of the war?	N	Mean	SD	*t*-value	*p*-value
Psychological	Yes	204	2.76	0.64	1.906	0.058
	No	83	2.60	0.64		
Economical	Yes	204	2.88	0.65	1.509	0.132
	No	83	2.75	0.66		
Social	Yes	204	3.40	0.61	2.499	0.013
	No	83	3.20	0.59		
Health	Yes	204	2.89	0.81	3.353	0.001
	No	83	2.57	0.68		
Family	Yes	204	3.23	0.55	3.381	0.001
	No	83	3.00	0.48		
Total Future Anxiety	Yes	204	2.99	0.50	3.264	0.001
	No	83	2.78	0.48		

**TABLE 6:** The below table shows the differences between those who have family who still lives in an environment of war and conflict and those who haven’t, in terms of future anxiety and its dimensions.

Dimensions	Does your family still live in an environment of war and conflict?	N	Mean	SD	*t*-value	*p*-value
Psychological	Yes	196	2.73	0.68	0.635	0.526
	No	91	2.68	0.57		
Economical	Yes	196	2.86	0.67	0.798	0.425
	No	91	2.80	0.63		
Social	Yes	196	3.35	0.59	0.414	0.679
	No	91	3.32	0.65		
Health	Yes	196	2.77	0.71	−0.815	0.417
	No	91	2.86	0.91		
Family	Yes	196	3.17	0.54	0.326	0.744
	No	91	3.15	0.53		
Total Future Anxiety	Yes	196	2.94	0.50	0.194	0.846
	No	91	2.92	0.50		

According to the table above, there is significant difference between those who have lived in Yemen during the war and conflicts and those who haven’t in terms of economical dimension (*t*-value = 2.775, *p*-value = 0.006), health dimension (*t*-value = 2.623, *p*-value = 0.009), and total future anxiety (*t*-value = 2.227, *p*-value = 0.027). All these differences were in favor of those who have lived in Yemen during the war and conflicts. On the other hand, the *t*-test showed insignificant differences between those who have lived in Yemen during the war and conflicts and those who haven’t, in terms of psychological, Social, and Family dimensions (*p*-value > 0.05).

According to the table above, there is significant difference between those who have been (or their family) exposed to any harm because of the war, and those who haven’t in terms of social dimension (*t*-value = 2.499, *p*-value = 0.013), health dimension (*t*-value = 3.353, *p*-value = 0.001), family dimension (*t*-value = 3.381, *p*-value = 0.001) and total future anxiety (*t*-value = 3.264, *p*-value = 0.001). All these differences favored those who have been (or their family) exposed to any harm because of the war. On the other hand, the *t*-test showed insignificant differences between those who have been (or their family) exposed to any harm because of the war and those who haven’t, in terms of psychological and economic dimensions (*p*-value > 0.05).

According to the table above, *t*-tests showed insignificant differences between those who have family still live in an environment of war and conflict and those who haven’t, in terms of future anxiety and its dimensions (*p*-values > 0.05).

## Discussion

Painful experiences in the environment of war and conflict and the psychological and social pressures that young people are exposed to in this environment may lead to frustration and fear of the future. Perhaps this is one of the biggest problems facing international students coming from such stressful and unstable environments. Several studies (Thabet et al., 2004; Montgomery, 2011; Pfeiffer & Elbert, 2011; Dimitry, 2012; Freh, 2016; Snoubar & MuSAH, 2017) indicate the spread of long-term psychological disorders among young people coming from areas that have witnessed wars and conflicts, the most important of which are depression and anxiety that may persist with young people even in a safe environment. Thus, this situation could hinder their adaptation in the host countries and affect their academic achievement. Thus, social work professionals must explore how the future anxieties and fears of war-and conflict-affected youth are affected by the continuation of wars in their home country. Understanding these concerns about the future of this vulnerable group of young people will provide the indicators and information needed to develop psychosocial support and social services programs to mitigate the impact of war and conflict on the mental health of young people and their future orientations.

This study aimed to determine the level of future anxiety among Yemeni international students coming from areas experiencing wars and conflicts by testing some hypotheses. The study results indicate that future anxiety was not significantly high among international students from war and conflict areas. This result is consistent with the results of a study conducted by Pavlova et al. (2020), which indicates that there is no clear relationship between the level of anxiety and the experience of living in an environment of war and conflict among young people and that these results can be explained that subjective feelings often affect feelings of anxiety. As for some demographic variables and their relationship to future anxiety, the study results showed a statistically significant relationship between demographic variables (economic status and academic year) and the level of future anxiety among students in the study sample. In this study, only economic status and academic year were associated with future anxiety levels among the sociodemographic variables. The results of this study agree with the study of Hamid & Mohamed (2014) and Salem (2015), where the results of their study showed the inverse relationship between the economic status variable and future anxiety among university students. The study results of Rabei et al. (2020) also indicate the effect of future anxiety on the performance of university students, and there is a statistically significant relationship between the academic year variable and the future anxiety of university students.

The current study indicated statistically significant differences between males and females (in favor of males) in the level of the economic dimension of future anxiety. There are statistically significant differences between males and females (in favor of females) in the level of the social dimension of future anxiety. Studies conducted in this context indicate the prevalence of psychological disorders such as anxiety among females more than males due to living in an environment of war and conflict (Murthy & Lakshminarayana, 2006). These results are consistent with the results of Thabet and Sultan (2016) study conducted on a sample of university youth victims of war and conflict, which indicates that there is a relationship between anxiety and sex of war-affected young and that exposure to previous traumatic events due to armed conflict has long-term negative effects on university students that differ according to their gender this may increase their mental health problems. Also this result is consistent with a study conducted on the relationship between COVID-19 and future anxiety. The results showed a close relationship between future anxiety and gender variables and occupational status; future anxiety among females is higher than in men (Dublaga and Grysztar, 2021). The current study results also indicated statistically significant differences between those who have been married and those who have never been married (in favor of those who have never been married) in the level of the economic dimension of future anxiety.

The current study results showed a statistically significant relationship between future anxiety and some variables related to war and conflict (living in a war environment - direct and indirect exposure to damage). There are statistically significant differences between those who lived in Yemen at the time of wars and those who did not live (in favor of those who lived in Yemen at the time of wars) in the level of future anxiety. There are also statistically significant differences between those exposed to harm or their family because of the war and those who were not exposed (in favor of those who were exposed) in the level of future anxiety as a whole. Living in an environment witnessing war and conflict directly affects feelings of fear, anxiety, and orientation towards the future due to the traumatic events that young people may witness or may be directly exposed to. Young people who have been exposed to stressful events and traumatic experiences differ from young people who have not lived in this environment in that they show symptoms related to mental health (Williams, 2006; McMullen et al., 2012; Williams et al., 2014; Pfefferbaum et al., 2019). Looking at research and studies that dealt with young people coming from war and conflict areas, we find that social support, security, culture, and education are among the most important areas that this group needs. To facilitate the adaptation of this category and improve their outlook on life and the future, there is an urgent need for guidance programs and programs that provide psychological and social support (Nakeyar et al., 2018; Thabet & Abdalla, 2018). The future anxiety of young people who have experiences of living in a war environment increases, and this is due to the nature of this stressful environment, which does not indicate a hopeful future due to the war’s destruction of property and infrastructure, it’s weakening of the economy and its impact on all community institutions, which makes predicting a positive future difficult for young people.

### Limitations of the Study

One limitation of this investigation is that more than half of the study population refused to participate in the research. It was also difficult to communicate with the females initially to do the clairvoyant interview due to cultural reservations. Also, the sample is limited and may not represent the most significant number of Yemeni students, so it is necessary to research the same topic on a larger sample so that the results can be generalizable.

### Implications for Social Work Practice

The study presents some useful indicators that can be used in social work with young people affected by conflicts and wars. The most important aspects that affect international students’ fears and future anxiety coming from these environments are outlined. There is an urgent need to provide psychosocial support services to this vulnerable group of young people to improve their future outlook (Nakeyar et al., 2018). To develop sustainable mental health, it is necessary to provide social support to young people affected by traumatic experiences. There is an interconnection between social support and mental health that may make these young people flexible in looking to the future and developing sustainable mental health (Törrönen, 2021). In this context, it is important to consider these students’ mental health, cultural, social, and economic dimensions in general and female students in particular and support them in obtaining psychosocial support services. Through the researcher’s good knowledge in this context, believes that it is necessary to use a female social worker in dealing with these students at the beginning to break some cultural barriers and encourage female students to benefit from psychosocial support services and to involve them in many other programs as the presence of such services mitigates discrimination against women coming from war zones, which may have witnessed many forms of racial discrimination.The university’s social work unit hosting international students needs to develop psychosocial support programs, especially for students from war and conflict areas. It may be helpful for the social worker to know the social support networks for these students and work with them to develop them. For the well-being of students affected by war and conflict, it is necessary to help them benefit from formal and informal social support networks. It may also be helpful to integrate these students with other international students who have had the same experiences through support groups.

## Conclusion

Studying the level of future anxiety among international students affected by war and conflict from a social work perspective is of great importance due to the increasing size of this vulnerable group of young people. The current study, through the indicators it provided, may contribute to strengthening the direct practice of social work in issues related to the fears of the future among young people in today’s society, in which crises accompanying wars, conflicts, and the global health crisis are growing, whose repercussions have affected the clarity of the future for young people. The importance of this study also stems from the fact that it focused on the future anxiety of young people coming from war and conflict areas, which were not given enough study and research in social work. Despite the quality of services provided by the host countries to international students, there is an urgent need to develop psychological and social support programs for students affected by wars and conflicts. In this context, social workers and researchers must have a good knowledge of the issues of discrimination against women coming from these societies and deal with them within the appropriate cultural framework, which facilitates the support of women and their receipt of services provided on the same level with males, which makes their outlook for the future more positive.

## Data Availability

The raw data supporting the conclusions of this article will be made available by the authors, without undue reservation.
